# The Enigma of the Tinnitus-Free Dream State in a Bayesian World

**DOI:** 10.1155/2014/612147

**Published:** 2014-07-06

**Authors:** Dirk De Ridder, Kathleen Joos, Sven Vanneste

**Affiliations:** ^1^Department of Surgical Sciences, Dunedin School of Medicine, University of Otago, P.O. Box 56, Dunedin 9054, New Zealand; ^2^BRAI²N & TRI, Sint Augustinus Hospital, Antwerp, Belgium; ^3^Department of Translational Neuroscience, Faculty of Medicine, University of Antwerp, Belgium; ^4^School of Behavioral and Brain Sciences, The University of Texas at Dallas, USA

## Abstract

There are pathophysiological, clinical, and treatment analogies between phantom limb pain and phantom sound (i.e., tinnitus). Phantom limb pain commonly is absent in dreams, and the question arises whether this is also the case for tinnitus. A questionnaire was given to 78 consecutive tinnitus patients seen at a specialized tinnitus clinic. Seventy-six patients remembered their dreams and of these 74 claim not to perceive tinnitus during their dreams (97%). This can be most easily explained by a predictive Bayesian brain model. That is, during the awake state the brain constantly makes predictions about the environment. Tinnitus is hypothesized to be the result of a prediction error due to deafferentation, and missing input is filled in by the brain. The heuristic explanation then is that in the dream state there is no interaction with the environment and therefore no updating of the prediction error, resulting in the absence of tinnitus.

## 1. Introduction

Fundamental concepts in psychology and philosophy of the mind are the notion of sensation and perception [1]. When a stimulus produces an effect on different sensory receptors it induces sensation. Subsequent interpretation and organization of this sensory stimulus produce a meaningful experience of the world and of one's perception [1]. Although in most cases perception is conscious, perception without awareness does exist, that is, the interpretation of semantic stimuli [2]. Normally wakefulness and awareness are related; one has to be awake; that is, there has to be a certain level of consciousness to be aware of something; that is, there is content in consciousness [3]. In states of deep sleep, anesthesia, and coma there is little or no wakefulness and hence no awareness. In drowsiness and light sleep there is more awareness. However, in certain states, dissociations exist between wakefulness and awareness, such as in the vegetative state, when there is wakefulness presumably without awareness (eyes open, brain shut) [4]. In the dream state there is awareness (content in consciousness) with decreased wakefulness (level of consciousness) [3]. Dreams are succession of images, ideas, emotions, and perceptions without sensations that occur involuntarily in the mind predominantly during rapid eye movement (REM) sleep.

Nonpulsatile subjective tinnitus is considered a phantom perception [5], the conscious awareness of a percept in the absence of an external stimulus. It is characterized by the perception that the phantom sound comes from an external sound source, even though the sound might be pulled from memory [1, 6, 7]. This is reminiscent of a dream state, when there is awareness, with stimuli attributed to the external world but generated internally [8]. Whereas tinnitus can be considered a simple phantom percept, dreams could be considered complex phantom percepts, like hallucinations and hallucinosis [9, 10]. However, in contrast to hallucinations and hallucinosis that occur during wakefulness, dreams occur during certain stages of sleep.

Stimulus-evoked auditory cortical activation does not necessarily produce conscious auditory perception [11], and auditory perception is possible in the absence of auditory input: more than 80% of people with normal hearing perceive phantom sounds when placed in a soundproof room [12]. Likewise, after limb amputation almost all people experience a phantom limb [13], whereas 70% suffer from severe phantom pain [13].

A clear clinical analogy exists between phantom pain and disabling tinnitus [1, 14, 15]. There are also parallels between the pathophysiology of tinnitus and pain [1], as well as in the treatment [16, 17]. However, there are also differences between tinnitus and pain. While physiological pain is mediated via nociceptive pathways, no analogous physiological tinnitus pathways exist. This could explain why commonly available analgesics that suppress acute physiological body pain are inefficient in ameliorating tinnitus [18]. Also, medications such as antiepileptics and antidepressants, which are effective in the treatment of neuropathic pain [18], tend to be ineffective for tinnitus [19].

Many to most (33–100%) patients who suffer from phantom limb percepts do not experience phantom limb percepts in a dream state [20–23]. This has been explained as follows [8, 21, 23]: neural representation of the body derives from sensory and proprioceptive feedback from the body. During sleep, when the brain/mind is actively kept offline, this sensory feedback is lacking. Moreover, during REM sleep and in the absence of external inputs, dreaming could activate a set of innate or early life spatial-temporal categories [8]. So if REM sleep is a state of protoconsciousness, that is, a contextually emergent property of self-sustaining systems, the self as it appears in REM sleep dreams is no longer affected by waking experiences because it feeds from an embodied and functionally intact body scheme [8, 21].

In view of the pathophysiological analogy between tinnitus and pain, it can be hypothesized that tinnitus is absent in the dream state as well. We therefore explored this in a group of 78 consecutive tinnitus patients attending the Multidisciplinary Tinnitus Research Initiative Clinic at the University of Antwerp. A recently proposed pathophysiological model of phantom sound based on a predictive brain concept with Bayesian updating [24] might explain why tinnitus is not perceived during dreaming.

## 2. Methods

### 2.1. Participants

Seventy-eight patients (57 males and 21 females) with chronic, nonpulsatile tinnitus were included in this study with an average age of 48.78 years (Sd = 12.87) and an average tinnitus duration of 5.74 years (Sd = 6.96). Thirty-five patients perceive noise-like tinnitus, while 43 patients experience pure tone tinnitus. Forty-three patients had bilateral tinnitus; 12 patients perceive tinnitus holocranially, 12 on the left side and 11 on the right side. Antwerp University Ethics Committee reviewed and approved the study. All patients signed an approved informed consent in order to enroll into the study.

### 2.2. Questionnaire

A questionnaire was created based on previous research in phantom limb pain and dreaming [20]. The first question asked whether the tinnitus patient recalled if they dreamed during the night (1), followed by the question whether in their dreams they perceive tinnitus (2).

## 3. Results

Of the 78 participating patients only 2 (2.56%) declared that they do not recall their dreams, while 76 (97.44%) do. Of those 76 patients that do recall their dreams 74 (97.73%) state that they do not perceive tinnitus while dreaming or are not aware of having tinnitus during sleep.

## 4. Discussion

People with tinnitus do not perceive tinnitus in their dreams analogous to what is reported for many phantom limb perceptions [21, 25]. Dreams and wakefulness are both associated with awareness, but in one state of awareness there is no tinnitus (dreams), whereas in the other (wakefulness) there is tinnitus.

**Figure 1:**
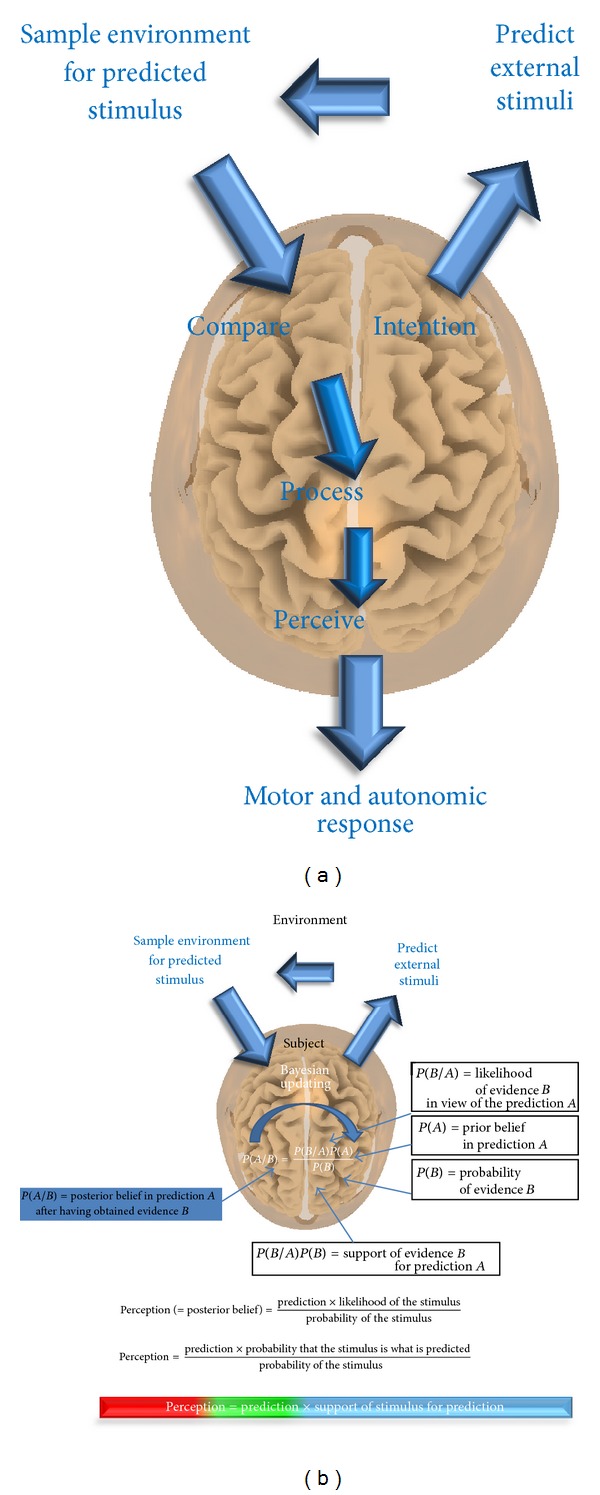
(a) The concept of the predictive brain; (b) the concept of Bayesian updating.

The reason why patients with tinnitus do not perceive tinnitus in their dream state can be theoretically explained by the Bayesian brain model which has been used as an explanation for the development of tinnitus in relation to auditory deafferentation [24]. This Bayesian brain model is founded on an extension of a predictive brain model (see Figure 1(a)).

Whereas other models (see [26] for an overview) can explain the tinnitus in the presence of deafferentation, they cannot explain why it would be absent in the dream state. The Bayesian model is compatible with both the deafferentation and noise-cancelling models [24] and provides a rationale why tinnitus develops in a wake state and not in a dream state. Previously proposed models rather describe how tinnitus would develop.

Physiologically the brain can be conceptualized as a Helmholtz machine [27] that constantly makes one or possibly multiple [28] predictions about the world. A Helmholtz machine tries to find a hidden structure in unlabeled data. Since the examples given to the learner are unlabeled, there is no error or reward signal to evaluate a potential solution; in other words, there is no updating of the predictions. A Bayesian brain however updates predictions based on what it actively explores in the environment by means of the senses [24, 29, 30]. Bayesian inference can therefore be conceptualized in a way that would be familiar to John Hughlings-Jackson as using sensory information from the environment to update memory-based expectations (held before acquiring sensory inputs) to produce posterior beliefs represented as percepts. This mechanism permits decision making based on predictions updated by actively sampling the environment for confirmation or rejection of expectations (see Figure 1(b)) [24].

Auditory deafferentation limits the amount of information the brain can acquire to make sense of the world. The topographically specific deafferentation induces a topographically specific prediction error hypothetically based on temporal incongruity [1]. In other words, it is inconsistent with what is stored in memory and should be updated. The model hypothesizes that deprived auditory information depends on the amount (bandwidth) of deafferented auditory channels [24]. Limited damage to auditory receptors causes loss of functional surround inhibition in the cortex, unmasking of latent inputs, and significantly altered neural coding. However, these changes do not lead to plasticity of the cortical map [31]. This suggests that the missing information can be obtained via access of overlapping tuning curves of the neighboring cortical cells. If the deafferentation is somewhat larger, a widening of auditory receptive fields [32] will permit pulling the missing information from the auditory cortical neighborhood. If this is insufficient, due to a still larger deafferentation, dendritic and axonal rewiring can occur [33]. If this is still insufficient, the missing auditory information can be pulled from (para)hippocampal memory [24].

**Figure 2:**
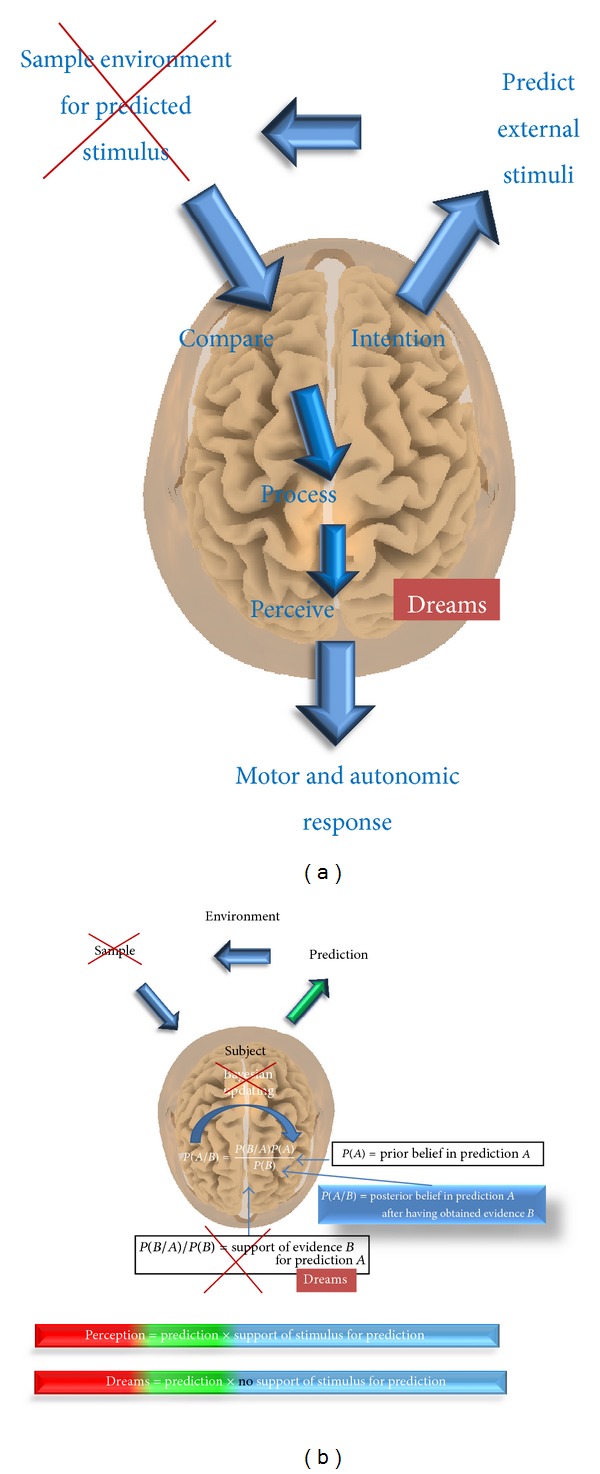
(a) Hypothetical explanation of the absence of tinnitus in dreams as seen from the predictive brain; (b) hypothetical explanation of the absence of tinnitus in dreams as seen from the Bayesian brain.

When we dream, we create an image of the world that is entirely detached from sensory feedback [34]; that is, it cannot be updated. This is under influence of decrease in monoamines in REM sleep. Aminergic activity is highest during waking, declines during NREM sleep, and is lowest during REM sleep. Cholinergic activity on the other hand shows the reverse pattern [34]. Sensory prediction errors are suppressed by aminergic influence during sleep [34]. This means that the discrepancy between top-down predictions and (the absence of) sensory signals received will not be registered, and the auditory deafferentation will not be filled in, resulting in the absence of tinnitus in the dream state (see Figures 2(a) and 2(b)) [26].

Indirect arguments for this hypothesis come from recent research on cerebellar influences in tinnitus. It has been argued that the cerebellum is involved in motor, sensory, and cognitive predictions [35]. It is therefore possible that auditory predictions are made in the paraflocculus, as removing this cerebellar structure can prevent tinnitus from arising and arrest the presence of tinnitus in animals [36]. This conceptually suggests that removing the prediction can prevent or abolish tinnitus, which is in accordance with the concept that tinnitus could be a malprediction [1].

However, apart from its theoretical implications, the data might also help to find the neural correlates of tinnitus. The putative on/off switch for tinnitus is to be found in these areas that differ between waking and REM state [26], that is, the ventrolateral prefrontal cortex/frontopolar-inferior parietal-cerebellar-parahippocampal network [10]. These areas overlap with a recent meta-analysis of PET studies in tinnitus [37] and provide a framework for zooming in on the pathophysiology of this enigmatic symptom.

In addition to its evident benefit for tinnitus research, it could also provide clues for consciousness research, by delineating the core areas involved in the neural correlates of consciousness; that is, minimal assembly of brain areas required for consciousness per se [38, 39].

Other potential explanations for the absence of tinnitus in the dream state have to be considered. It is possible that during the dream state there is an attention shift from the tinnitus to the dream, analogous to what is noted in patients who do not perceive their tinnitus when intensely engaged in a task.

In conclusion, this report demonstrates that tinnitus perception is switched off during dream sleep even though there is awareness, like in wakefulness. This suggests that it is theoretically possible to find the neural correlates of phantom sound and thereby find a potential avenue for suppressing this enigmatic symptom.
